# Influence of Internet-Based Health Management on Control of Clinical Parameters in Patients With Hypertension: Four-Year Longitudinal Study

**DOI:** 10.2196/42896

**Published:** 2023-03-20

**Authors:** Botian Chen, Yuqi Dou, Xue Yu, Defu Ma

**Affiliations:** 1 School of Public Health Peking University Health Science Center Beijing China

**Keywords:** hypertension, internet-based health management, blood pressure control, longitudinal study, health management, primary care, online based, eHealth, telehealth, telemedicine

## Abstract

**Background:**

In recent years, more and more studies have shown that internet-based health management can help patients with hypertension control their blood pressure. However, there is a lack of similar research in China.

**Objective:**

We designed this study to clarify the impact of long-term internet-based health management on the control of clinical parameters in patients with hypertension. These results are also expected to identify the relevant factors affecting the control of clinical parameters in hypertension more accurately toward developing more targeted health management strategies.

**Methods:**

This was a longitudinal study of internet-based health management in the five provinces of northwest China. The inclusion criteria were aged ≥18 years and no serious cognitive disease or mental disorder. After collecting the physical examination data of 8567 people in the five northwest provinces in 2013, we conducted online health management (including diet, exercise, and behavior) and follow-up. In the physical examination in 2013, 1008 new patients with hypertension were identified, who were divided into a good blood pressure control group and poor blood pressure control group. Physical examination and a questionnaire survey were conducted every 2 years to understand the changes of health management on the subjects’ health-related behaviors. We then analyzed the changes of clinical indicators related to hypertension and the influencing factors related to blood pressure control in patients with hypertension. All statistical analyses were performed using R software (version 4.1.2) and a *P* value <.05 was considered statistically significant.

**Results:**

A total of 8567 people met the inclusion criteria and underwent health management. Self-comparison showed that after 4 years of health management, the smoking cessation rate and amount of exercise significantly increased (both *P*<.001). The low-density lipoprotein-cholesterol levels also increased (*P*=.005), whereas the high-density lipoprotein-cholesterol levels decreased (*P*=.007). The newly discovered patients with hypertension in 2013 were further screened. After 4 years of health management, their smoking cessation rate increased significantly (*P*=.03) and the amount of exercise increased but not significantly (*P*=.08). In terms of clinical indicators, the diastolic blood pressure considerably decreased (*P*<.001) and the systolic blood pressure slightly decreased (*P*=.13). The correlation analysis of blood pressure control in patients with new-onset hypertension showed that gender (female) and changing relevant factors according to health management behaviors (BMI; cereals and potatoes intake; fish, livestock meat, and eggs intake; fruit intake; and physical activity) were the protective factors of blood pressure control.

**Conclusions:**

Internet-based health management has a significant and long-term effect on blood pressure control in patients with hypertension.

## Introduction

Hypertension is a worldwide health problem with increasing rates of impaired functional status, morbidity, disability, and mortality [1]. Recent studies have shown that people with comorbidities, including hypertension, are at a greater risk of severe COVID-19 [2]. According to global statistics, an estimated 1.39 billion (range 1.34-1.44 billion) adults aged ≥20 years worldwide have hypertension [3]. According to Chinese statistics, 23.2% (approximately 244.5 million) of adults have hypertension and 41.3% (approximately 435.3 million) of adults have prehypertension [4]. Due to the serious complications, hypertension remains the leading cause of death globally, accounting for 10.4 million deaths per year [5]. As a chronic disease, there is no clear method to cure hypertension. Fortunately, as the main cause of death in patients with hypertension is complications, control of the blood pressure (BP) in a safe range can effectively reduce the incidence of complications and thus greatly extend the life expectancy of patients. Therefore, controlling BP has become the main means to deal with hypertension [6,7].

Health management refers to the process of comprehensively managing the health risk factors of individuals or groups. The purpose of health management is to mobilize the enthusiasm of individuals and groups, and effectively use limited resources to achieve the maximum health effect [8]. In addition to medication, previous studies have shown that face-to-face health management can improve the lifestyle of patients with hypertension and in turn help to control their BP [9-12]. However, previous research results also pointed out that the effect of health management is difficult to maintain in the absence of continuing guidance [13]. The internet is the first source of medical information for the public and patients because of its speed and cost-effectiveness. Therefore, the internet and health management should be combined to achieve rapid and efficient results [14]. Recently, increasing studies have suggested that internet-based health management may be a useful replacement method to improve compliance. Good compliance is very important for the effective BP control of patients with hypertension [15]. Mano [16] showed that internet-based health management is quite effective. Furthermore, Baer et al [17] showed that internet-based health management can effectively improve weight control in patients with obesity. However, there is a lack of similar research on the BP control from internet-based health management in China.

Therefore, we designed this study to clarify the impact of long-term internet-based health management on the control of clinical parameters in patients with hypertension. This study is also expected to help identify the most relevant factors affecting the control of clinical parameters in patients with hypertension more accurately toward achieving targeted health management.

## Methods

### Research Design

This was a prospective, nonrandomized, longitudinal study performed in five provinces of northwest China. The inclusion criteria were aged ≥18 years and no serious cognitive disease or mental disorder. The exclusion criteria were aged <18 years with severe cognitive impairment or mental illness. By 2013, 56,542 people from Shaanxi, Gansu, Ningxia, Inner Mongolia, and Shanxi provinces, including 15 cities and 61 counties (districts, banners), had joined the health management platform, which is for personal use only. This platform includes modules such as exercise management and diet management. Complete relevant data were available for 51,486 people. The included study population participated in an annual medical checkup and completed a questionnaire every 2 years.

After collecting the physical examination data of the study participants in 2013, they received internet-based health management and follow-up. The platform is mainly divided into three modules: “understanding my health,” “improving my health,” and “health improvement effectiveness.” Physical examinations and the questionnaire were administered after 2 years (in 2015) and 4 years (in 2017). Hypertension was based on systolic blood pressure (SBP)≥140 mmHg and/or diastolic blood pressure (DBP)≥90 mmHg on repeated clinical measurements or a previous history of hypertension.

Based on the physical examination in 2013, we divided the newly identified patients with hypertension into two groups according to good and poor BP control. Good BP control was determined according to SBP<140 mmHg and DBP<90 mmHg on repeated clinical measurements; otherwise, the individual was considered to have poor BP control [18].

### Personal Health Management Platform

The health management platform is for private use only. Anyone can log in to their own account to access health records on the platform according to each physical examination to help users understand their own health status. Relevant experts then formulate an individualized diet, exercise, and other relevant opinions for individuals according to their health status, and carry out health management. After 3 months of health management, the personal health status is further assessed. Health records include individual basic information (eg, age, gender, height, weight) and clinical indicators (eg, BP, blood glucose, blood lipids) from the physical examination in that year, along with family history, diet, and exercise.

### Exercise Management

The system automatically formulates 12 stages of progressive exercise prescription according to the health status of an individual. Users can choose relevant sports as recommended and manually record the time and date of sports. One week later, the rationality of the recommendations is evaluated by comparing the exercise performed in the previous week with the recommended exercise prescription.

### Diet Management

The system platform automatically formulates meal prescriptions for individuals based on the submitted health questionnaire information. Users who want to create personalized recipes according to their personal dietary preferences only need to modify and replace the foods in the expert-recommended recipes. By recording at least 2 days of meal diaries every week, the system can analyze the dietary nutrition status, identify potential problems in meals, and make appropriate recommendations.

### Data Source

The research data were derived from the physical examination data of participants from the five provinces in northwest China and from the questionnaire survey. The health management for participants included dietary guidance; physical exercise guidance; living habits guidance; basic knowledge of hypertension, diabetes, and hyperlipidemia; and other aspects.

### Study Variables and Measurements

Basic characteristics, including age, sex, and family history of hypertension, were collected at the first physical examination. Individual parameters were collected by questionnaire after each physical examination, including health-related behaviors (smoking, drinking, physical activity, sedentary time [hours], sleeping time [hours], quality of sleep [very good, fair, not good, very bad]), dietary intake (cereals and potatoes; fish, livestock, poultry, meat, and eggs; milk and dairy; soybean and nuts; vegetables; fruit). Physical activity was rated according to the International Physical Activity Questionnaire scale [19]. Dietary intake was rated according to the 2016 Chinese dietary guidelines [20], divided into lower than the recommended intake, recommended intake, and higher than the recommended intake. The psychological status of participants was evaluated by the General Health Questionnaire (GHQ-12) [21]. Compared with the results in 2013, the improvement of the results in 2017 was considered to indicate an improvement in relevant risk behaviors following the health management plan.

The clinical measurement results of participants were imported into an epidata database after each physical examination. An automatic sphygmomanometer was used to measure the BP of the left arm (the measurement position was flush with the heart) in a sitting position after the subject rested quietly for at least 5 minutes. The measurement was repeated at intervals of at least 5 minutes and the average of the two readings was recorded. Venous blood samples were collected during fasting, and fasting blood glucose was measured by the glucose oxidase method according to routine operating procedures. Total cholesterol, triglyceride, and other blood lipid indicators were measured on an empty stomach. All the onsite measurements including laboratory tests were validated for internal quality control according to clinical standards.

### Ethical Considerations

This study was ethically reviewed and approved by the Biomedical Ethics Committee of Peking University (IRB00001052-0816). All participants in the study provided informed consent. All data were anonymized. No subsidy was provided to the participants.

### Statistical Analysis

Descriptive analysis was used to describe the basic characteristics of participants. The raw data were processed using the 99-quantile capping method to remove outliers. Analysis of variance was used to analyze the annual changes in the clinical indices of 8576 subjects and newly identified patients with hypertension. The *χ^2^* test or analysis of variance was used to analyze the annual changes of health-related behavior and diet as appropriate. Patients with new-onset hypertension were divided into groups according to their BP control after 4 years of health management, and the factors that may affect control were evaluated by the *χ^2^* test. Backward elimination was used to find the influencing factors of hypertension control and a binary logistic model was established. This model was selected since BP control is more meaningful than a cure for chronic diseases such as hypertension. All statistical analyses were performed on R software (version 4.1.2) and a *P* value <.05 was considered statistically significant. If the area under the receiver operating characteristic (ROC) curve was greater than 0.5, the result was considered to be meaningful.

## Results

### Basic Characteristics of the Cohort From Five Provinces in Northwest China in 2013

The baseline survey included 51,486 individuals, 42,347 (82.25%) of whom were healthy and 9139 (17.75%) of whom had hypertension (new onset+current onset). Among the healthy population, there was a slight majority of men. Among the patients with hypertension, there was a large majority of men (>80%). The average age of the patients with hypertension was higher than that of healthy participants. In addition, the intake of milk, fruits, and vegetables in the population at baseline was deemed to be seriously insufficient (Table 1).

**Table 1:** Basic characteristics of all subjects undergoing a physical examination in 2013.

Characteristics			Total (N=51,486)	Healthy (n=42,347)	Hypertension (n=9139)
**Demographic factors**					
	Age (years), mean (SD)		36.80 (8.66)	36.02 (8.22)	40.4 (9.58)
	**Gender, n (%)**				
		Man	32,408 (62.95)	25,026 (59.10)	7382 (80.77)
		Woman	19,078 (37.05)	17,321 (40.90)	1757 (19.23)
	**Family history of hypertension (father), n (%)**				
		Yes	6367 (12.37)	4694 (11.08)	1673 (18.31)
		No	45,119 (87.63)	37,653 (88.92)	7466 (81.69)
	**Family history of hypertension (mother), n (%)**				
		Yes	6611 (12.84)	4849 (11.45)	1762 (19.28)
		No	44,875 (87.16)	37,498 (88.55)	7377 (80.72)
**Health-related behaviors**					
	**Tobacco use, n (%)**				
		Nonsmoking	32,628 (63.37)	28,142 (66.46)	4487 (49.10)
		Current smoking	17,287 (33.59)	13,135 (31.02)	4152 (45.43)
		Quit smoking	1570 (3.04)	1070 (2.52)	500 (5.47)
	**Alcohol consumption, n (%)**				
		No alcoholic beverages	34,013 (66.06)	28,878 (68.19)	5135 (56.19)
		Drinking	17,473 (33.94)	13,469 (31.81)	4004 (43.81)
	**Physical activity (IPAQ^a^), n (%)**				
		Low	15,256 (29.63)	12,612 (29.78)	2644 (28.93)
		Medium	23,722 (46.07)	19,623 (46.34)	4099 (44.85)
		High	12,508 (24.30)	10,112 (23.88)	2396 (26.22)
	Sedentary time (hours), mean (SD)		5.23 (2.75)	5.27 (2.76)	5.04 (2.74)
	**Cereals and potato intake, n (%)**				
		Below the recommended intake	21,631 (42.01)	18,134 (42.82)	3497 (38.26)
		Moderate to recommended intake	15,381 (29.87)	12,686 (29.96)	2695 (29.49)
		Higher than recommended intake	14,474 (28.12)	11,527 (27.22)	2947 (32.25)
	**Fish, livestock meat, and eggs intake, n (%)**				
		Below the recommended intake	26,009 (50.52)	21,543 (50.87)	4466 (48.87)
		Moderate to recommended intake	12,747 (24.76)	10,466 (24.71)	2281 (24.96)
		Higher than recommended intake	12,730 (24.72)	10,338 (24.42）	2392 (26.17)
	**Milk and dairy products intake, n (%)**				
		Below the recommended intake	45,585 (88.54)	37,420 (88.37)	8165 (89.34)
		Moderate to recommended intake	5901 (11.46)	4927 (11.63)	974 (10.66)
	**Soybeans and nuts intake, n (%)**				
		Below the recommended intake	33,943 (65.92)	27,883 (65.84)	6060 (66.31)
		Moderate to recommended intake	2864 (5.56)	2351 (5.55)	513 (5.61)
		Higher than recommended intake	14,679 (28.52)	12,113 (28.61)	2566 (28.08)
	**Vegetables intake, n (%)**				
		Below the recommended intake	38,980 (75.71)	32,166 (75.96)	6814 (74.56)
		Moderate to recommended intake	8545 (16.60)	6967 (16.45)	1578 (17.27)
		Higher than recommended intake	3961 (7.69)	3214 (7.59)	747 (8.17)
	**Fruit intake, n (%)**				
		Below the recommended intake	44,682 (86.78)	36,567 (86.35)	8115 (88.80)
		Moderate to recommended intake	5038 (9.79)	4276 (10.10)	762 (8.34)
		Higher than recommended intake	1766 (3.43)	1504 (3.55)	262 (2.86)
**Sleep and psychological condition**					
	**Quality of sleep, n (%)**				
		Very good	11,442 (22.22)	9483 (22.39)	1959 (21.44)
		Fair	31,761 (61.69)	26,208 (61.89)	5553 (60.76)
		Not good	6840 (13.29)	5498 (12.98)	1342 (14.68)
		Very bad	1443 (2.80)	1158 (2.74)	285 (3.12)
	Sleeping time (hours), mean (SD)		7.23 (1.16)	7.25 (1.14)	7.18 (1.21)
	Psychological score^b^, mean (SD)		17.77 (5.05)	17.72 (5.04)	17.98 (5.05)

^a^IPAQ: International Physical Activity Questionnaire.

^b^A higher score indicates a worse psychological state.

### Changes in Health-Related Behaviors and Clinical Indicators of Eligible Participants

Of all subjects screened, a total of 8567 individuals screened in 2013, 2015, and 2017 were deemed to be eligible for inclusion in the study. After 4 years of health management, the smoking cessation rate increased significantly (*P*<.001), the amount of exercise increased significantly (*P*<.001), and other health-related behaviors also improved to varying degrees. The improvement of various behaviors showed a trend of strengthening with the increase of health management time. In terms of clinical indicators, low-density lipoprotein cholesterol levels of the participants increased (*P*=.005) and high-density lipoprotein cholesterol levels decreased (*P*=.007), although the change of indicators was small, which may be related to their age. There was no significant difference in other clinical indices measured over time. The awareness rate of hypertension among eligible participants in 2013 was 26.48% (Table 2). After 4 years of internet-based health management, the awareness rate of hypertension among this population increased significantly to 30.82%. The control rate of hypertension showed the same increasing trend. In 2013, the control rate of patients with hypertension was only 6.13%, which significantly increased to 22.61% after 4 years of health management.

**Table 2:** Changes of self-management and clinical parameters of eligible participants (N=8567).

Parameter		2013	2015	2017	*P* value
**Self-management indices**					
	Quit smoking^a^, n (%)	249 (7.90)	357 (10.80)	391 (11.48）	<.001
	Sufficient exercise, n (%)	5324 (62.15)	5512 (64.34)	5604 (65.41)	<.001
	Sufficient cereal and potato intake, n (%)	4789 (55.90)	4851 (56.62)	4861 (56.74)	.49
	Sufficient fish, eggs, poultry, and livestock meat intake, n (%)	4418 (51.57)	4475 (52.24)	4524 (52.81)	.27
	Sufficient milk and dairy intake, n (%)	1106 (12.91)	1112 (12.98)	1125 (13.13)	.91
	Sufficient soybean and nut intake, n (%)	4537 (52.96)	4519 (52.75)	4541 (53.01)	.94
	Sufficient vegetable intake, n (%)	1986 (23.18)	1961 (22.89)	1979 (23.10)	.90
	Sufficient fruit intake, n (%)	874 (10.20)	898 (10.48)	902 (10.53)	.75
	Times of drinking per week, mean (SD)	0.916 (1.716）	0.927 (1.693)	0.937 (1.680)	.73
**Clinical parameters, mean (SD)**					
	SBP^b^ (mmHg)	116.08 (12.89)	116.41 (12.91)	116.41 (13.20)	.32
	DBP^c^ (mmHg)	76.87 (10.38)	76.90 (10.33)	76.71 (10.48)	.39
	FPG^d^ (mmol/L)	5.42 (2.99)	5.38 (2.79)	5.35 (2.64)	.38
	TC^e^ (mmol/L)	5.14 (4.49)	5.06 (4.30)	4.96 (3.94)	.09
	TG^f^ (mmol/L)	2.11 (2.29)	2.08 (0.22)	2.13 (2.19)	.36
	LDL-C^g^ (mmol/L)	1.85 (1.41)	1.91 (1.36)	1.92 (1.34)	.005
	HDL-C^h^ (mmol/L)	2.05 (1.89)	1.99 (1.82)	1.96 (1.69)	.007
	BMI (kg/m^2^)	23.09 (4.05)	23.10 (3.75)	23.16 (3.73)	.21

^a^The smoking cessation rate was calculated as smoking in the year/total number of smokers in that year.

^b^SBP: systolic blood pressure.

^c^DBP: diastolic blood pressure.

^d^FPG: fasting plasma glucose.

^e^TC: total cholesterol.

^f^TG: triglyceride.

^g^LDL-C: low-density lipoprotein cholesterol.

^h^HDL-C: high-density lipoprotein cholesterol.

### Changes in Health-Related Behaviors and Clinical Indices of Patients With New-Onset Hypertension

In the physical examination of 2013, 1008 new patients with hypertension were found. After 4 years of health management, their smoking cessation rate increased significantly (*P*=.03), the amount of exercise increased slightly (*P*=.08), and other health-related behaviors also improved to varying degrees. The improvement of various behaviors also showed the trend of strengthening with the increase of health management time. In terms of clinical indicators, the DBP decreased considerably (*P*<.001) and the SBP decreased slightly (*P*=.13). However, there was no significant change detected in other clinical indices. The improvement of various indicators showed a trend of continuous improvement with an increase in health management time (Table 3).

**Table 3:** Changes of health-related behaviors and clinical parameters in patients with new-onset hypertension (N=1008).

Parameter		2013	2015	2017	*P* value
**Health-related behaviors**					
	Quit smoking^a^, n (%)	32 (5.27)	85 (8.34)	94 (9.18)	.03
	Sufficient physical activity (IPAQ^b^), n (%)	631 (62.60)	665 (65.97)	678 (67.26)	.08
	Sufficient cereal and potato intake, n (%)	551 (54.66)	546 (54.17)	553 (54.86)	.95
	Sufficient fish, eggs, poultry, and livestock intake, n (%)	503 (49.90)	516 (51.19)	522 (51.79)	.69
	Sufficient milk and dairy intake	145 (14.38)	137 (13.59)	150 (14.88)	.71
	Sufficient soybean and nut intake, n (%)	530 (52.58)	517 (51.29)	522 (51.79)	.84
	Sufficient vegetable intake, n (%)	237 (23.51)	228 (22.62)	224 (22.22)	.78
	Sufficient fruit intake, n (%)	110 (10.91)	117 (11.61)	111 (11.01)	.87
	Times of drinking per week, mean (SD)	1.09 (2.23)	1.09 (2.20)	1.08 (2.15)	>.99
**Clinical parameters, mean (SD)**					
	SBP^c^ (mmHg)	127.54 (16.45)	127.11 (16.15)	126.10 (16.24)	.13
	DBP^d^ (mmHg)	91.96 (8.77)	89.68 (9.98)	88.55 (10.70)	<.001
	FPG^e^ (mmol/L)	6.01 (3.79)	5.94 (3.70)	5.89 (3.57)	.75
	TC^f^ (mmol/L)	5.88 (5.88)	5.73 (5.71)	5.60 (5.58)	.54
	TG^g^ (mmol/L)	2.75 (2.99)	2.63 (2.82)	2.63 (2.75)	.56
	LDL-C^h^ (mmol/L)	2.04 (1.75)	2.06 (1.67)	2.06 (1.65)	.94
	HDL-C^i^ (mmol/L)	2.34 (2.38)	2.26 (2.31)	2.20 (2.24)	.43
	BMI (kg/m^2^)	24.69 (5.33)	24.60 (5.09)	24.58 (4.92)	.87

^a^The smoking cessation rate was smoking in the year/total number of smokers in that year.

^b^IPAQ: International Physical Activity Questionnaire.

^c^SBP: systolic blood pressure.

^d^DBP: diastolic blood pressure.

^e^FPG: fasting plasma glucose.

^f^TC: total cholesterol.

^g^TG: triglyceride.

^h^LDL-C: low-density lipoprotein cholesterol.

^i^HDL-C: high-density lipoprotein cholesterol.

### Related Factors of BP Control in Patients With New-Onset Hypertension

After 4 years of health management in the patients with new-onset hypertension (N=1008), 195 (19.35%) patients had good BP control and 813 (80.65%) patients had poor BP control. Statistical analysis showed that younger patients had better control than older patients (*P*=.05) and women achieved better control than men (*P*=.03). In addition, those who changed their intake of cereals and potatoes, or fish, poultry, meat, and eggs according to the guidance of health management achieved better BP control (*P*<.001). As shown in Table 4, in comparison with patients who did not follow health management guidance, those who changed their milk and dairy intake or fruit intake according to health management guidance achieved better BP control (*P*<.001). Improving physical activity was also found to be beneficial for BP control in this population (*P*<.001). Moreover, reducing sedentary time emerged as a favorable factor in reducing BP (*P*=.01). Those who improved their mental state according to the guidance of health management also had better BP control (*P*=.004). There were no significant differences in the remaining indicators according to good or poor BP control at the end of the study period (Table 4).

**Table 4:** Factors related to blood pressure control in patients with new-onset hypertension.

Factors			Total (N=1008)	Well- controlled (<140/90 mmHg) (n=195)	Poorly controlled (≥140/90 mmHg) (n=813)	*P* value
**Demographic factors**						
	Age (years), mean (SD)		37.23 (8.43)	36.15 (7.89)	37.48 (8.54)	.05
	**Gender, n (%)**					.03
		Male	791 (78.5)	141 (17.8)	650 (82.2)	
		Female	217 (21.5)	54 (24.9)	163 (75.1)	
	**Family history of hypertension (father), n (%)**					.77
		Yes	149 (14.8)	27 (18.1)	122 (81.9)	
		No	859 (85.2)	168 (19.6)	691 (80.4)	
	**Family history of hypertension (mother), n (%)**					.27
		Yes	143 (14.2)	33 (23.1)	110 (76.9)	
		No	865 (85.8)	162 (18.7)	703 (81.3)	
**Change in behavioral factors according to the guidance of health management, n (%)**						
	**Smoking**					.13
		Yes	637 (63.2)	133 (20.9)	504 (79.1)	
		No	371 (36.8)	62 (16.7)	309 (83.3)	
	**Drinking**					.83
		Yes	673 (66.8)	132 (19.6)	541 (80.4)	
		No	335 (33.2)	63 (18.8)	272 (81.2)	
	**Cereals and potatoes intake**					<.001
		Yes	343 (34.0)	87 (25.4)	256 (74.6)	
		No	665 (66.0)	108 (16.2)	557 (83.8)	
	**Fish, eggs, poultry, and livestock intake**					<.001
		Yes	364 (36.1)	99 (27.2)	265 (72.8)	
		No	644 (63.9)	96 (14.9)	548 (85.1)	
	**Milk and dairy products intake**					<.001
		Yes	114 (11.3)	36 (31.6)	78 (68.4)	
		No	894 (88.7)	159 (17.8)	735 (82.2)	
	**Soybeans and nuts intake**					.07
		Yes	184 (18.3)	45 (24.5)	139 (75.5)	
		No	824 (81.7)	150 (18.2)	674 (81.8)	
	**Vegetable intake**					.94
		Yes	677 (67.2)	130 (19.2)	547 (80.8)	
		No	331 (32.8)	65 (19.6)	266 (80.4)	
	**Fruit intake**					<.001
		Yes	166 (16.5)	51 (30.7)	115 (69.3)	
		No	842 (83.5)	144 (17.1)	698 (82.9）	
	**Physical activity (IPAQ^a^)**					<.001
		Yes	500 (49.6)	120 (24.0)	380 (76.0)	
		No	508 (50.4)	75 (14.8)	433 (85.2)	
	**Sedentary time**					.01
		Yes	112 (11.1)	32 (28.6)	80 (71.4)	
		No	896 (88.9)	163 (18.2)	733 (81.8)	
	**Sleeping time**					.77
		Yes	791 (78.5)	151 (19.1)	640 (80.9)	
		No	217 (21.5)	44 (20.3)	173 (79.7)	
	**Sleeping quality**					.09
		Yes	797 (79.1)	145 (18.2)	652 (81.8)	
		No	211 (20.9)	50 (23.7)	161 (76.3)	
	**Psychological state**					.004
		Yes	426 (42.3)	101 (23.7)	325 (76.3)	
		No	582 (57.7)	94 (16.2)	488 (83.8)	

^a^IPAQ: International Physical Activity Questionnaire.

### Factors Related to BP Control in Patients With New-Onset Hypertension

To clarify the influencing factors of hypertension control, we performed regression analysis. Binary logistic regression analysis showed a significant correlation between health-related behavior and the ability to achieve BP control in patients with hypertension. Achieving BP control was more difficult for patients who did not follow the guidance of health management to change fish, livestock, poultry, meat, and egg intake; fruit intake; increase physical activity; and improve their psychological state. The area under the ROC curve was 0.6787 (Table 5).

**Table 5:** Binary logistics model of factors related to blood pressure control after health management in patients with hypertension.

Variable		Estimate (B)	aOR^a^ (95% CI)	*P* value
Intercept		–1.6612	N/A^b^	<.001
Female gender (male=reference)		–0.3079	0.73 (0.50-1.09)	.12
Age (years)		0.0155	1.02 (1.00-1.04)	.14
**No change according to the guidance of health management (change=reference)**				
	BMI	0.299	1.35 (0.97-1.89)	.08
	Cereal and potato intake	0.333	1.40 (0.99-1.95)	.05
	Fish, eggs, poultry, and livestock intake	0.5328	1.70 (1.21-2.39)	.002
	Fruit intake	0.4829	1.62 (1.08-2.41)	.02
	Physical activity (IPAQ^c^)	0.4776	1.61 (1.16-2.26)	.005
	Psychological state	0.3783	1.46 (1.05-2.02)	.02

^a^aOR: adjusted odds ratio.

^b^N/A: not applicable.

^c^IPAQ: International Physical Activity Questionnaire.

## Discussion

### Principal Findings

Long-term internet-based health management has a good effect on the BP control of patients with hypertension, especially on regulating SBP. Moreover, this control effect is long-lasting and not easy to rebound. In addition, this study found that being young; female; more likely to follow the guidance of health management to control BMI within a reasonable range; adjust intake levels of cereals and potatoes, fish, eggs, poultry, livestock, milk, and fruit; and appropriately increase physical activity have a significant effect on the control of BP in patients with hypertension.

After 4 years of health management, 195 (19.35%) of the 1008 patients with hypertension had achieved good BP control. In 2018, the BP control rate of Chinese patients with hypertension (age≥18 years) was reported to be much lower at only 11.0% [22], demonstrating a potential benefit of health management. McManus et al [23] also showed that internet-based health management has a good effect on BP control in patients with hypertension. In addition, Hu et al [13] showed that face-to-face health management can effectively help patients to control their BP during the implementation of management, but rebound readily occurs after the end of management. However, due to the long-term nature of internet-based health management, this rebound can be effectively avoided.

This study found that changing dietary intake, appropriately increasing physical activity, adjusting the mental state, and controlling BMI according to health management guidance can effectively increase the control rate of BP in patients with hypertension. In addition, the rate of BP control was higher in female patients and in younger patients. Atik et al [24] and Li et al [25] showed that a lower BMI helps to control BP in patients with hypertension. Shim et al [26] and Duchame-Smith et al [27] pointed out that diet may be an important factor in BP control in patients with hypertension. Some bioactive ingredients may explain the cardioprotective effects of diet, such as vitamins, essential elements, dietary fiber, and phytochemicals [28-31]. The potential mechanisms of action may involve antioxidation; anti-inflammation; and regulation of blood sugar, blood lipids, and BP [32,33]. These previous results are consistent with the findings of this study.

### Strengths and Limitations

Strengths of our study include the consistency of the findings in this study and other cohort studies, together with the fact that internet-based health management has strong universality. Moreover, we combined logistic regression to analyze the related factors of BP control in patients with hypertension and also analyzed dietary components in more detail than in previous studies.

However, some limitations should be noted. To date, this research has been carried out over a relatively short time and therefore the longer-term effects are unknown; however, our research will continue to clarify the effect of internet-based health management on BP control in patients with hypertension. In addition, our study population was only recruited from five provinces in northwest China, which is still small compared with the whole country. However, the incidence rate of hypertension in these provinces represents the medium level for nationwide statistics [34], which can better represent the whole country. In the future, we will try our best to implement internet-based health management across the country. In addition, we did not assess changes of treatment rate. Although treatment rate is very important in chronic disease research, this was beyond the focus of this study. Moreover, many environmental factors can influence BP measurement [35,36]. However, in this study, all participants were sitting in a quiet environment to measure the BP of the left arm (the measurement position was flush with the heart), and the difference in BP caused by measurement error between participants was small. Our clinical parameters were self-reported by the subjects according to the physical examination in that year. Although this may cause some bias, the parameters we collected are relatively conventional and therefore the bias is not expected to be substantial. Another limitation is that our study only focused on the influence of dietary behavior factors on BP, without considering drug factors. Finally, we have not evaluated some new promising biomarkers of cardiovascular risk prediction and atherosclerosis, such as high-sensitivity C-reactive protein, fibrinogen, matrix metalloproteinases, and myeloperoxidase [37].

### Conclusion

Our findings confirm that internet-based health management has a significant effect on BP control in patients with hypertension, which can be maintained over the long-term. However, at present, the attention to internet-based health management remains insufficient, and there is still a long way to go to comprehensively popularize this management strategy.

## Abbreviations

BP: blood pressure
DBP: diastolic blood pressure
GHQ-12: General Health Questionnaire
ROC: receiver operating characteristic
SBP: systolic blood pressure

## Data Availability

The data sets generated during and analyzed during the current study are not publicly available due to data confidentiality requirements but are available from the corresponding author on reasonable request.
